# The Sequence (S) index as a marker of diminished step-to-step transition efficiency in older adults

**DOI:** 10.3389/fnhum.2026.1710840

**Published:** 2026-02-25

**Authors:** Elham Alijanpour, Ashwini Kulkarni, Peter G. Adamczyk, Daniel M. Russell

**Affiliations:** 1 Ellmer College of Health Sciences, Old Dominion University, Norfolk, VA, United States; 2 College of Engineering, University of Wisconsin-Madison, Madison, WI, United States

**Keywords:** aging, energy loss, gait efficiency, ground reaction forces, S index, step-to-step transition

## Abstract

**Introduction:**

Age-related declines in walking efficiency are often attributed to musculoskeletal and neuromuscular changes, yet the mechanisms underlying these inefficiencies remain poorly understood. One key contributor is the step-to-step transition (STST), during which the center of mass (CoM) is redirected between limbs. The Sequence (S) index quantifies the temporal overlap between push-off and collision forces during STST, with higher values indicating greater mechanical energy loss. This study investigated whether aging affects STST efficiency, as quantified by the S index, and examined the timing and impulse characteristics underlying observed differences.

**Methods:**

Thirteen young and eleven older healthy adults walked at five speeds relative to their preferred walking speed on an instrumented treadmill while ground reaction force data were collected.

**Results:**

Older adults exhibited significantly higher S index values across all speeds, indicating less efficient gait. These differences were accompanied by shorter pre-HC duration, lower total push-off impulse, lower single support push-off impulse, and higher double support collision impulse. Both groups showed reductions in S index at higher speeds, primarily through increased single-support timing and impulses, and decreased double-support overlap, but older adults remained less efficient overall.

**Discussion:**

These results suggest that aging impairs the temporal and mechanical coordination of STST. This effect may potentially be due to neuromuscular changes. The S index offers a step-level, mechanically grounded metric for assessing gait efficiency and may provide insight into energetic cost in older populations.

## Introduction

Age-related declines in walking efficiency have been attributed to multiple physiological changes including musculoskeletal decline, neuromuscular deterioration, and altered motor control (Boyer et al., 2023; Chastan et al., 2009; Mian et al., 2006; Seidler et al., 2010). Among the mechanisms contributing to reduced walking efficiency with age, step-to-step transition (STST), the process by which the center of mass is redirected between limbs during the transition from one step to the next, is a major factor (Donelan et al., 2002a). Recent evidence indicates that STST is disrupted in older adults (Alijanpour and Russell, 2025; Chong et al., 2009), yet the specific characteristics underlying these age-related changes remain poorly understood.

STST is process by which the center of mass (CoM) is redirected from one leg to the other during gait (Donelan et al., 2002a; Ruina et al., 2005). During the stance phase of gait, the body CoM behaves as an inverted pendulum, conserving mechanical energy by requiring minimal active work to sustain forward progression. At the end of terminal stance, however, the CoM reaches the limit of this arc and must be redirected to initiate the next step. This redirection is generated by push-off from the trailing leg, and marks the onset of STST (Adamczyk and Kuo, 2009). During STST, positive mechanical work is produced through push-off and negative work is absorbed during the collision as the leading leg contacts the ground. Energy efficiency is reduced when push-off and collision occur simultaneously, as the positive work from push-off partly cancels out the negative work from the collision leg (Ruina et al., 2005). Ruina et al. (2005) demonstrated that the relative timing of push-off and collision strongly influences walking efficiency. Inefficient overlap results in energy loss and can be quantified using the sequence (S) index. The S index reflects the degree to which collision precedes or overlaps with push-off, with higher values indicating greater temporal overlap and increased energetic loss. This metric was originally termed “overlap parameter, S_O_” (Adamczyk and Kuo, 2009; Ruina et al., 2005). We adopted “sequence index, S” since Ruina et al. (2005) identified sequencing of leg forces as a specific strategy for reducing locomotion costs, and S index is a metric to quantify this sequencing. *Overlap parameter* describes what is measured (temporal overlap of forces). *Sequence Index* describes what the measure represents biomechanically (how forces are sequenced across limbs). The terminology change improves clarity by making explicit the underlying biomechanical mechanism, while maintaining the identical mathematical definition from prior work.

The S index quantifies the overlap between push-off and collision. *An S index of 0 indicates complete separation of push-off and collision, representing theoretically minimal energy loss* (i.e., push-off is completed before collision). An S index of 0.5 reflects simultaneous push-off and collision, leading to greater energy loss. An S index of 1.0 reflects collision fully preceding push-off leading to maximum energy loss. Thus, smaller S index values reflect more efficient sequencing, where the trailing leg completes push-off and generates forward and upward momentum before the leading leg begins absorbing the CoM’s energy during loading response. This timing facilitates a smoother and more efficient redirection of CoM. In younger adults, the push-off predominantly occurs before the leading-limb collision, resulting in a low S index [approximately 0.047 described as S_o in Adamczyk and Kuo (2009)]. This separation minimizes the simultaneous performance of positive (push-off) and negative (collision) work, thereby conserving energy.

In younger adults, STST begins before the initial contact of the front leg and ends after the toe-off of the trailing leg, resulting in minimal temporal overlap between push-off and collision (Adamczyk and Kuo, 2009). In contrast, older adults initiate STST at leading-limb’s contact and complete it at trailing-limb toe-off, suggesting greater overlap between push-off and collision (Chong et al., 2009). Less efficient STST has been linked to increased energy expenditure, shorter step length, and reduced walking speed (Chong et al., 2009; Ruina et al., 2005), all of which are well-documented age-related changes in gait of older adults (Boyer et al., 2023). At a given walking speed, older adults expend approximately 31% more metabolic energy than younger adults despite similar total mechanical work, suggesting that while energy-saving mechanisms may be preserved with age, overall walking efficiency declines (Mian et al., 2006). The theoretical (Ruina et al., 2005) and empirical evidence (Chong et al., 2009) suggests that quantifying STST efficiency using the S index in older adults can reveal how older adults manage the mechanical and temporal demands of STST. Understanding these age-related changes is essential, as inefficient STST may underlie slower preferred walking speeds and elevated energetic cost in older adults.

In a recent study, we found that while overall coordination patterns during gait remain largely similar between young and older adults, aging affects lower extremity coordination particularly with delayed and earlier transitions during initiation and completion of STST, respectively (Alijanpour and Russell, 2025). The altered STST timing in older adults coincides with well-documented age-related declines in ankle torque (Thelen et al., 1996), propulsive power (Franz, 2016), and proprioceptive feedback (Goble et al., 2009; Shaffer and Harrison, 2007). Emerging evidence also highlights age-related structural changes in the midbrain (Hu et al., 2023) and dopaminergic pathways (Chastan et al., 2009), neural systems critical for motor planning and temporal coordination (Hu et al., 2023; Seidler et al., 2010), as potential contributors to altered STST in aging (Chastan et al., 2009). However, it remains unclear whether age-related alterations in STST reflect limitations in neuromuscular capacity, or adaptive recalibrations of motor control strategies optimized under age-related constraints. Distinguishing between these possibilities s critical, as it has substantial implications for intervention design and expected outcomes (Jeon et al., 2025; Mathieu et al., 2024; Summerside et al., 2024).

**Figure 1:**
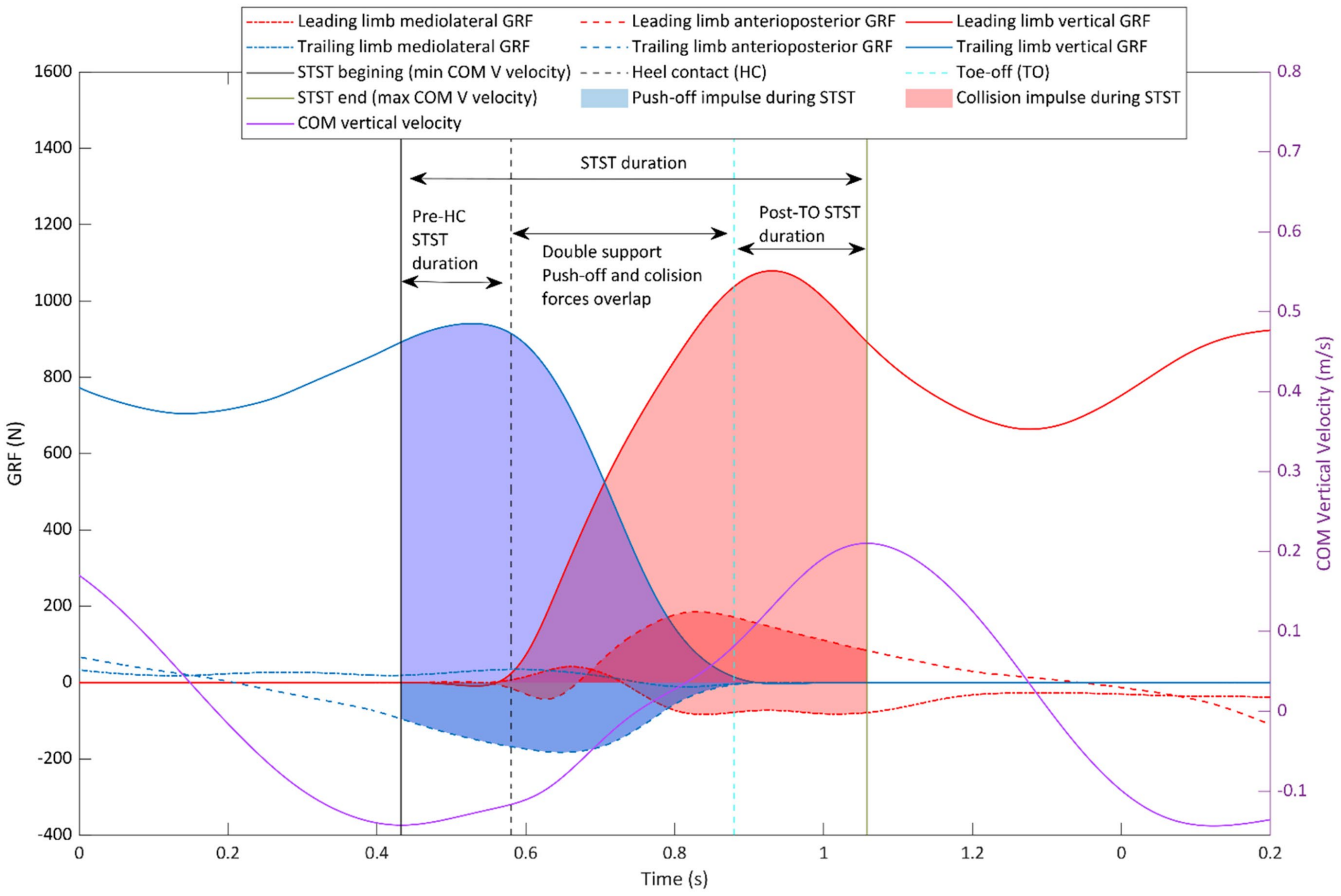
Illustration of step-to-step transition (STST) characteristics, beginning with the minimum vertical velocity of the center of mass and ending at its maximum. The efficiency of STST can be improved, and energy loss reduced, by minimizing the overlap between push-off and collision forces (spending less time during double support). The purple solid line represents vertical velocity of center of mass and the solid black and green vertical lines indicate minimum of COM vertical velocity (STST beginning) and maximum of COM vertical velocity (STST end), respectively. Blue lines represent 3D ground reaction forces for the trailing limb and the blue shaded region shows the area underneath the trailing limb 3D GRF (impulse). Red lines represent 3D ground reaction forces for the leading limb and the red shaded region shows the area underneath the leading limb 3D GRF (impulse). The dashed black vertical line represents leading limb heel contact and dashed blue vertical line shows trailing limb toe-off, the area between these two vertical lines represents the double support and push-off and collision forces overlap.

Figure 1 illustration of the characteristics of STST and the variables influencing the S index. STST begins when the vertical velocity of CoM reaches its minimum and ends when it reaches its maximum. The S index quantifies the temporal overlap between the 3D ground reaction forces (GRF) generated by the trailing (push-off) and leading (collision) legs during STST, with greater overlap indicating higher S index values and increased energetic loss (Adamczyk and Kuo, 2009).

Possible mechanical changes that could reduce the S index include:

1. Earlier initiation of STST prior to leading-limb heel contact (HC), thereby starting the transition during single-leg push-off and reducing temporal overlap with collision.
2. Prolonging STST completion beyond trailing limb toe-off, allowing collision to occur predominantly during single-support.
3. Increasing push-off and collision impulses during single support, which reduces reliance on double support force exchange.
4. Decreasing push-off and collision impulses during double support, thereby minimizing overlap between positive and negative mechanical work.

This study compares leg forces sequencing (quantified using S index) between young and older adults across five walking speeds relative to their PWS, aiming to understand how aging influences gait efficiency and energy loss during STST. We hypothesized that older adults would exhibit higher S index values than younger adults across all speeds, and that the S index would decrease (i.e., approach to 0) with increasing walking speed, likely due to greater push-off demands. Because the S index reflects a complex interaction of timing and impulse strategies, we further analyzed STST using timing and impulse variables. Timing variables included: (a) total STST duration, given that push-off and collision impulses are integrated over the entire transition window, (b) STST duration before heel contact (pre-HC STST), because earlier STST initiation reduces the overlap between push-off and collision forces, (c) STST duration following toe-off (post-TO STST), because prolonged STST completion may reduce the overlap between push-off and collision forces, and (d) percentage of STST spent in double support, since greater double support duration increases force overlap. Impulse variables included: (a) total push-off and collision impulses during STST, (b) single support push-off and collision impulses during STST as higher single support impulses reduce the overlap between push-off and collision forces, and (c) double support push-off and collision impulses as higher double support impulses increase the overlap between push-off and collision forces. This framework provides a detailed assessment of the timing and impulse mechanisms underlying the observed S index values and enables evaluation of age-related strategies used to achieve lower S index values at higher walking speeds.

## Methods

### Participants

Thirteen young adults (females = 6, age = 25.38 ± 3.04 years (mean ± SD), height = 1.72 ± 0.07 m, weight = 75.64 ± 12.27 kg, PWS = 1.29 ± 0.11 m/s) and eleven older adults (females = 7, age = 71.70 ± 4.18 years, height = 1.72 ± 0.16 m, weight = 71.70 ± 11.88 kg, PWS = 1.28 ± 0.16 m/s) participated in this study (see Appendix A. Table 1) for detailed demographic information. Participants had no recent history of major musculoskeletal injury, falls or surgery; no lower extremity arthritis or joint pain, no cardiovascular or neurological pathology, and a body mass index of less than 30 kg/m^2^. The local institutional Review Board approved the study. Written informed consent was obtained from all participants, and the study was conducted in accordance with the Declaration of Helsinki.

### Experimental setup

Data collection for each participant was conducted in a single session. Participants were asked to walk across a 6.1-meter instrumented mat (ProtoKinetics, USA) five times to determine their overground PWS instructed as the comfortable speed they use for daily activities. After preparation, each participant performed 3 min of walking on an instrumented treadmill at five different speeds (80, 90, 100, 110, and 120% of the PWS). Nexus software 2.16 (Vicon Co, Oxford, UK) was used to record GRF data.

### Data processing

GRF data were low pass filtered with a 4th-order Butterworth filter at a 20 Hz cut-off (Mai and Willwacher, 2019). Vertical GRFs were used to define initial contact and toe-off points. CoM vertical acceleration and velocity were calculated using a combined leg forces method (Donelan et al., 2002b). Local minima and maxima of the CoM vertical velocity were identified to define the onset and end of STST, respectively. The S index was calculated following Adamczyk and Kuo (2009), using STST timing, along with push-off and collision forces for the last minute of each trial. For each participant and walking speed, a custom MATLAB script was used to remove the crossover steps on the dual-belt instrumented treadmill to ensure the accuracy of push-off and collision forces. This algorithm identifies the foot placement using the heel and medial toe markers and retained only steps in which foot remained on its ipsilateral belt of the treadmill throughout the stance phase. To calculate S index, first, the cumulative fraction of the net push-off and net collision impulses were computed prior to time *t* within the STST (Equations 1, 2). Where $P_{\mathrm{PO}}^{*}$ and $P_{\mathrm{CO}}^{*}$ are the net push-off and collision impulses, respectively, and ${\hat{P}}_{\mathrm{PO}}^{*}$ and ${\hat{P}}_{\mathrm{CO}}^{*}$ are the direction vector of the net push-off and collision impulses. Finally, the S index is calculated by the integral of the collision fraction with respect to the push-off fraction (Equation 3, Adamczyk and Kuo, 2009). The average S index will be calculated for statistical analysis.


$$\mathrm{qPO}(t)=\frac{P_{\mathrm{PO}}(t).{\hat{P}}_{\mathrm{PO}}^{*}}{\left\|P_{\mathrm{PO}}^{*}\right\|}$$ (1)

$$\mathrm{qCO}(t)=\frac{P_{\mathrm{CO}}(t).{\hat{P}}_{\mathrm{CO}}^{*}}{\left\|P_{\mathrm{CO}}^{*}\right\|}$$ (2)

$$s_{0}=\int_{0}^{1}\text{qPOdqPO}$$ (3)

To characterize factors contributing to variation in S index, we quantified both timing and impulse variables that influence its value. STST duration was calculated by subtracting the time of maximum CoM vertical velocity from the time of minimum COM vertical velocity for each step. Pre-HC STST was computed by subtracting heel contact time from STST start time, with negative values indicating STST begins before heel contact. Post-TO STST was computed by subtracting toe-off time from STST end time, with positive values indicating STST extends beyond toe-off. Another key measure was the percentage of STST spent during double support (from leading leg heel contact to trailing leg toe-off), where a higher value leads to greater force overlap. For impulse variables, single support push-off and collision impulses, and double support push-off and collision impulses, all normalized to body weight and integrated over time, were computed (Deffeyes and Peters, 2021). Greater single-support impulses indicate reduced temporal overlap between push-off and collision, whereas greater double-support impulses indicate increased overlap. In addition, total push-off and collision impulses during STST were calculated (Figure 1).

Step length and step width were calculated using the position of ipsilateral and contralateral heel markers at each ipsilateral step as recommended method for treadmill walking (Supakkul, 2017). The step rate was calculated as the number of steps per minute for the last minute of each trial.

### Statistical analysis

We addressed three primary statistical questions: (a) whether outcome variables differed between age groups (young vs. older adults), (b) whether outcome variables differed across walking speeds (list all speeds here), and (c) whether the effects of walking speed on outcome variables differed between age groups (age × speed interaction). To answer these questions, PWS was first compared between age groups to confirm non-inferiority of baseline walking speed. Multilevel linear modeling (MLM) was used to examine the effects of age groups, walking speed, and their interaction on the S index, STST timing variables, and impulse variables. MLM was selected to account for the repeated-measures structure of the data, as each participant completed several trials across walking speeds, resulting in non-independent observations and potentially unequal number of observations across individuals. For each outcome variable (S index, STST timing, and impulse variables), models included fixed effects of covariates for age group (older vs. young), walking speed (80%, 90, 100, 110, and 120% of PWS), and their interaction. Participant identity was included as a random intercept to account for individual differences in baseline outcome levels. Random slopes for walking speed were included when supported by the data.

All models were estimated with restricted maximum likelihood (REML). Statistical analyses were conducted using SPSS (IBM SPSS Statistics 25, SPSS Inc., Chicago, IL). In each model, walking speed (five levels) was treated as a within-subjects covariate, and age group (young, older) as a between-subjects covariate. To systematically address study aims, five specific statistical questions were evaluated for each dependent variable:

1. Does the dependent variable vary significantly across individuals? (to justify and confirm the choice of MLM statistical analysis)

This initial step evaluated whether meaningful between-participant variability existed, thereby justifying the use of MLM. Intraclass correlation coefficient (ICC) between the residuals obtained from the within-subject and between-subject variance analysis were calculated. An ICC greater than 0.05 was considered indicative of meaningful clustering at participant level (i.e., repeated measures nested within participants), justifying the use of MLM to account for the hierarchical structure of the data and potential non-independence of observations (Lorah, 2018). Since this test was significant and the ICC exceeded 0.05, we could proceed with MLM to answer the abovementioned scientific questions.

2 Does age group difference explain variations in the outcome variable? [to answer whether outcome variables are significantly different between age groups (young vs. older adults)]

This tested the main effect of age groups as a covariate on each outcome variable. Both the repeated-measures and random intercept covariance structures were specified as *scaled identity* reflecting equal variance and no assumed correlation among repeated observations at this stage.

3 Does linear change in speed explain the variations in the outcome variable? (to answer whether an increase in walking speed can significantly affect the outcome variables)

This tested the main effect of walking speed as a covariate, assuming a linear trend across the five walking speeds. The linear trend assumption was based on observed group-level means across conditions. The same *scaled identity* covariance structure was applied for both random intercept and repeated measures in this model.

4 Does the effect of walking speed vary across individuals? (to investigate any moderation in the effect of speed)

This model evaluated whether participants differed in their response to increasing walking speed by testing random slopes for speed. The random intercept structure was modeled as *unstructured*, and the repeated-measures structure was modeled as *scaled identity* to allow for participant-specific trends. If this model was significant, meaning the behavior of outcome variable due to changes in speed differs between participants, then we would proceed to the following model to explore the interaction between age and speed.

5 Does the relationship between speed and the outcome variable differ by age group? (to answer whether there is an interaction between the effects of age and walking speed)

This tested the age group × speed interaction. For this model, the repeated-measures covariance structure was specified as *first-order autoregressive (AR1)* to account for correlations between adjacent speeds, and the random intercept structure was modeled as *unstructured*.

Statistical significance was evaluated using Type III tests of fixed effects, and the alpha level was set at *p* < 0.05 for all analyses. Effect sizes were quantified using a proportional reduction in residual variance (pseudo-*R*^2^), calculated as the proportional reduction in level-1 residual variance (statistical question 1) when adding the predictor of interest to the model (Equation 4). Specifically, pseudo-*R*^2^ was computed as (Lorah, 2018):

$$R^{2}=\frac{\varepsilon_{\mathrm{ij}}(m1)-\varepsilon_{\mathrm{ij}}(m2)}{\varepsilon_{\mathrm{ij}}(m1)}$$ (4)

Where $\varepsilon_{\mathrm{ij}}(m1)$ and $\varepsilon_{\mathrm{ij}}(m2)$ denote the residual variance from the reduced (statistical question 1) and full models (statistical questions 2, 3, or 4), respectively. Following recommended guidelines for multilevel models (Lorah, 2018), pseudo-*R*^2^ values of approximately 0.02, 0.15, and ≥0.35 were interpreted as small, medium, and large effects, respectively, consistent with adapted benchmarks from Cohen (1992).

## Results

### Walking speed and gait parameters

There was no significant difference in walking speeds between young and older adults, [*t*(22) = −0.22, *p* = 0.82, *b* = −0.01], with a mean difference of 0.014 m/s (across all five conditions), well below the 0.1 m/s threshold for comparable speeds defined by Boyer et al. (2017). However, a non-inferiority test using a 0.1 m/s margin was inconclusive, as the 95% confidence interval (CI, −0.13 to 0.10) crossed the margin, preventing a conclusive claim of non-inferiority (see Appendix A. Table 2 for participant-specific walking speeds). To further examine age-related differences in the S index persisted when young and older adults were compared at strictly matched absolute speeds, we conducted a supplementary analysis in which participants walked at seven fixed speeds ranging from 0.5 to 1.7 m/s. Only seven participants in each age group were able to complete all matched-speed trials; therefore, this supplementary analysis was limited to S-index outcomes and followed identical processing and analysis procedures. Results from this matched-speed analysis are reported in Appendix B.

**Figure 2:**
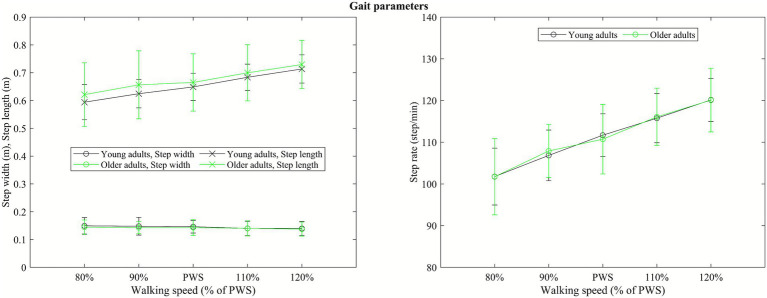
Illustration of group average step length and step width (left side panel), and step rate (right side panel) at each walking speed.

There was no significant difference in step rate (95% CI: −5.32 to 5.44 step/min), step length (95% CI: −0.03 to 0.08 m), and step width (95% CI: −0.02 to 0.02 m) between younger and older adults (*p*’s > 0.05, Figure 2).

### S index

**Figure 3:**
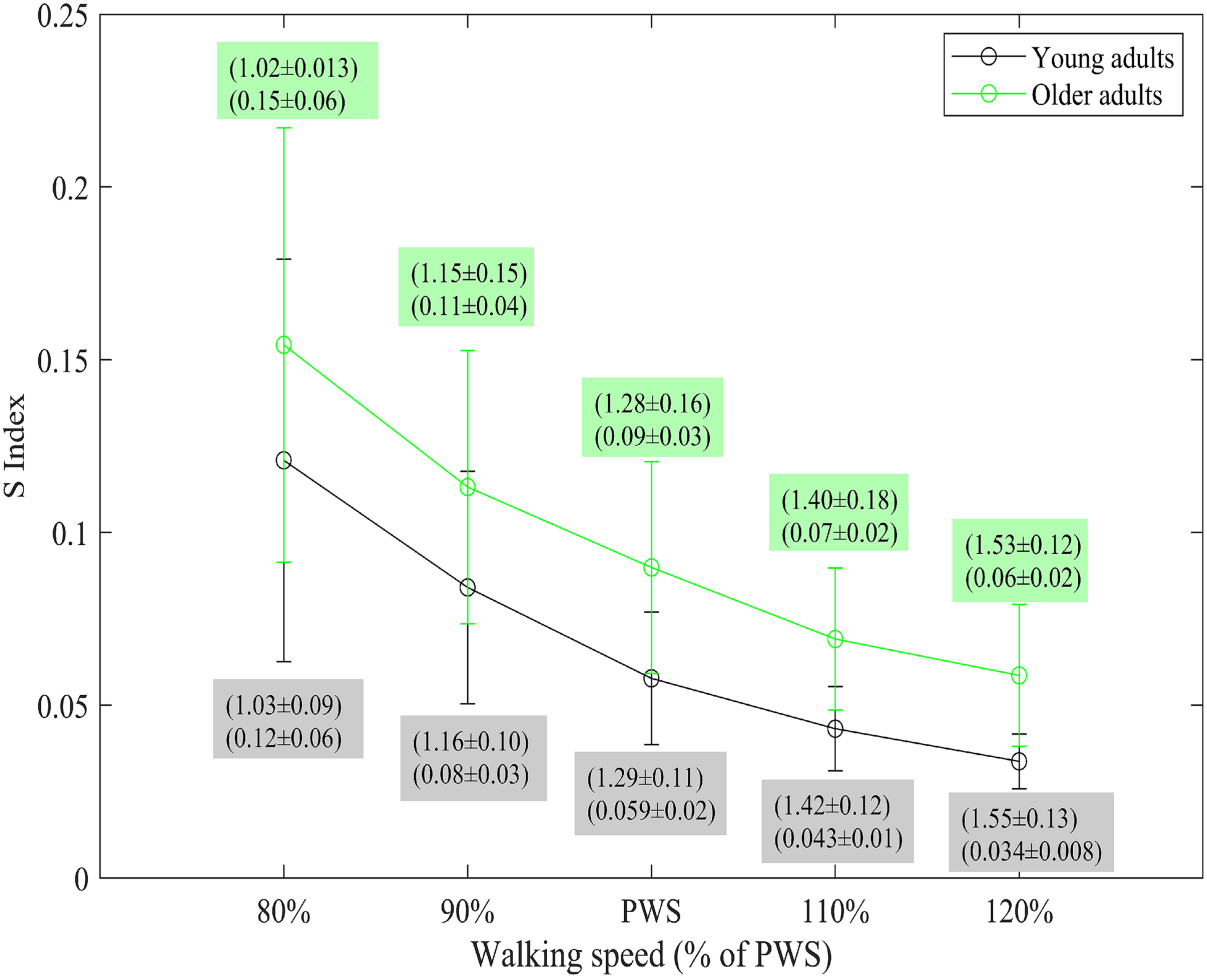
Mean ± SD for the S index values. Data for young (black) and older adults (green) walking at five different speeds relative to their preferred walking speed (PWS) is presented. The error bars indicate 1 SD. Mean ± 1 SD of the walking speed (top values in each box) and S index (bottom values in each box) in each group for young (green text box) and older adults (grey text box) are depicted for each speed condition.

Older adults exhibited 0.03 BW.s higher S index compared to younger adults, *t*(22) = 2.50, *p* = 0.02, *R*^2^ = 0.30, *b* = 0.03, indicating a medium effect size. The S index significantly decreased with increasing speed, with a change rate of 0.022 per approximately 0.13 m/s increase in speed, *t*(95) = −14.73, *p* < 0.001, *R*^2^ = 0.69, *b* = −0.022, representing a strong positive negative linear relationship between walking speed and S index. Age group did not significantly influence the linear relationship between speed and S index, *t*(35.95) = −0.06, *p* = 0.95 (Figure 3, see Appendix A. Table 3 for participant-specific values). Importantly, an additional analysis at matched absolute speeds (0.5–1.7 m/s) confirmed that older adults consistently exhibited higher S index values than young adults across all speeds (see Appendix B). This replication indicates that the observed age-related differences are not dependent on scaling speeds to PWS and supports the robustness of our main findings.

### STST timing variables

**Figure 4:**
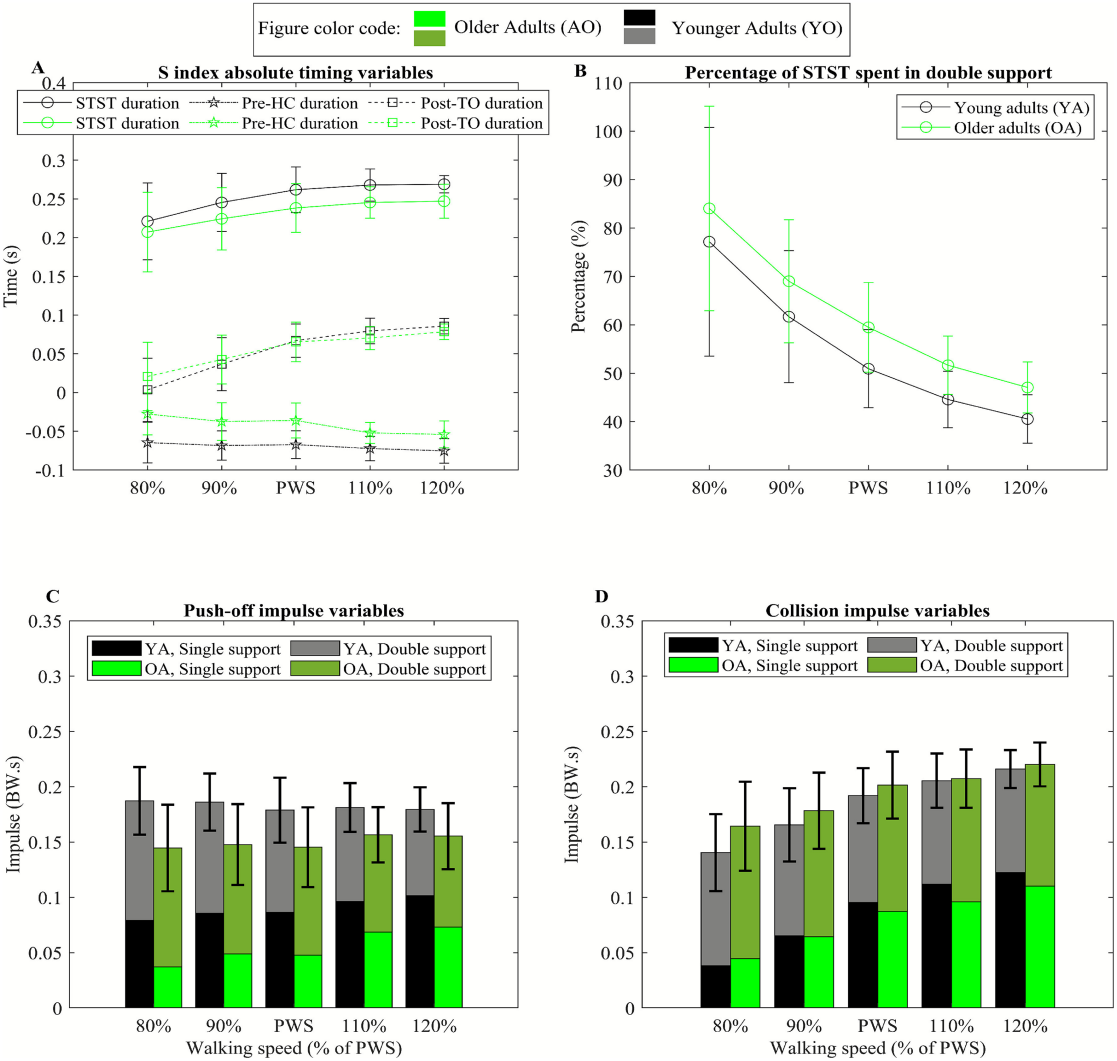
Black and green color represent the group average for young adults (YA) and older adults (OA), respectively. **(A)** The panel A represent step-to-step transition (STST) timing variables where circles show STST total duration, the squares indicate pre heel contact (pre-HC) STST duration, and the stars illustrate post toe-off (post-TO) STST duration, at five different speeds based on PWS. The error bars indicate ±1 standard deviation. **(B)** Circles represent percentage of STST time spent in double support at five different speeds based on preferred walking speed (PWS). The error bars indicate ±1 standard deviation. **(C)** Panel C represents push-off impulse variables where bottom part of the bars indicates single support impulse and top part of the bars shows double support impulse at five different speeds based on PWS. The height of the bars illustrates total impulse. The error bars indicate ±1 standard deviation of total impulse. **(D)** Panel D represents collision impulse variables where bottom part of the bars indicates single support impulse and top part of the bars shows double support impulse at five different speeds based on PWS. The height of the bars illustrates total impulse. The error bars indicate ±1 standard deviation of total impulse.

There was no significant difference in STST duration between younger and older adults, *t*(22) = −1.83, *p* = 0.08, *b* = −0.021. STST duration significantly increased with increasing speed, with a change rate of 0.01 s per approximately 0.13 m/s increase in speed, *t*(95) = 7.75, *p* < 0.001, *R*^2^ = 0.38, *b* = 0.01, indicating a strong positive linear relationship between speed and STST duration. Age group did not significantly moderate the relationship between speed and STST duration, *t*(36.10) = −0.17, *p* = 0.86 (Figure 4A).

Older adults exhibited approximately 0.03 s shorter pre-HC STST duration compared to younger adults, *t*(22) = 3.88, *p* < 0.001, *R*^2^ = 0.41, *b* = 0.028, indicating a large effect of age. Pre-HC STST duration significantly increased with walking speed, at a rate of 0.004 s per approximately 0.13 m/s increase in speed, *t*(95) = −5.94, *p* < 0.001, *R*^2^ = 0.26, *b* = −0.004, reflecting a moderate linear relationship between speed and pre-HC STST duration. Age groups did not significantly influence this relationship, *t*(22.01) = 1.68, *p* = 0.11 (Figure 4A).

There was no significant difference in post-TO STST duration between younger and older adults, *t*(22) = 0.11, *p* = 0.91. Post-TO STST duration significantly increased with walking speed with a change rate of 0.02 s per approximately 0.13 m/s increase in speed, *t*(95) = 13.97, *p* < 0.001, *R*^2^ = 0.67, *b* = 0.018, indicating a strong linear relationship between speed and post-TO STST duration. However, age groups did not significantly moderate the linear relationship between speed and post-TO STST duration, *t*(31.63) = 1.57, *p* = 0.12 (Figure 4A).

There was no significant difference in percentage of STST spent in double support between younger and older adults, *t*(22) = 4.20, *p* = 0.10. The percentage of STST spent in double support significantly decreased with increasing walking speed, with a reduction of 9.08% per approximately 0.13 m/s increase in speed, *t*(95) = −15.76, *p* < 0.001, *R*^2^ = 0.72, *b* = −9.08, reflecting a strong negative linear relationship between speed and percentage of STST spent in double-support. Age group did not significantly moderate this relationship, *t*(34.03) = 0.04, *p* = 0.97 (Figure 4B).

### STST impulse variables

Older adults exhibited 0.03 (BW.s) lower total push-off impulse during STST compared to young adults, *t*(22) = −3.04, *p* = 0.006, *R*^2^ = 0.27, *b* = −0.034, indicating a medium effect of age. There was no significant difference in double support push-off impulse during STST between young and older adults, *t*(22) = 0.32, *p* = 0.75. However, older adults exhibited approximately 0.03 (BW.s) lower single-support push-off impulse compared to younger adults, *t*(22) = −3.79, *p* < 0.001, *R*^2^ = 0.40, *b* = −0.03, indicating a large age effect. There was no significant linear effect of speed on total push-off impulse, *t*(95) = 0.78, *p* = 0.43. Double-support push-off impulse significantly decreased with increasing walking speed, at a rate of 0.007 (BW.s) per approximately 0.13 m/s increase in speed, *t*(95) = −26.02, *p* < 0.001, *R*^2^ = 0.88, *b* = −0.007, reflecting a very strong negative linear relationship. Single-support push-off impulse significantly increased with increasing walking speed, at a rate of 0.007 (BW.s) per approximately 0.13 m/s increase in speed, *t*(95) = 8.46, *p* < 0.001, *R*^2^ = 0.42, *b* = 0.007, reflecting a very strong positive linear relationship. Age groups did not significantly affect the linear trend between speed and push-off impulses, *p* > 0.05 (Figure 4C).

There was no significant difference in total collision impulse during STST between young and older adults, *t*(22) = 0.96, *p* = 0.35. Older adults exhibited 0.02 (BW.s) higher double support collision impulse during STST compared to young adults, *t*(22) = 2.73, *p* = 0.01, *R*^2^ = 0.22, *b* = 0.017, reflecting a medium effect of age. There was no significant difference in single-support collision between younger and older adults, *t*(22) = −0.63, *p* = 0.54. Total collision impulse significantly increased with walking speed, at a rate of 0.02 (BW.s) per approximately 0.13 m/s increase in speed, *t*(95) = 17.15, *p* < 0.001, *R*^2^ = 0.75, *b* = 0.017, reflecting a strong positive relationship. Double-support collision impulse significantly decreased with increasing walking speed, at a rate of 0.002 (BW.s) per approximately 0.13 m/s increase in speed, *t*(95) = −8.92, *p* < 0.001, *R*^2^ = 0.45, *b* = −0.002, reflecting a strong negative relationship. Single-support collision impulse significantly increased with walking speed, at a rate of 0.02 (BW.s) per approximately 0.13 m/s increase in speed, *t*(95) = 19.21, *p* < 0.001, *R*^2^ = 0.79, *b* = 0.019, indicating a strong positive relationship. Age groups did not significantly affect the linear trend between speed and collision impulses, *p* > 0.05 (Figure 4D).

## Discussion

This study investigated age-related changes in STST efficiency by comparing S index, a measure of temporal overlap between push-off and collision, in young and older adults. Our results demonstrated that older adults exhibited a consistently higher S index (lower efficiency-higher energy loss) with a medium effect size across all speeds compared to young adults. To understand underlying mechanisms contributing to the age-related higher S index, we further examined STST timing and impulse characteristics, allowing us to identify specific motor control and mechanical factors contributing to gait inefficiency with aging. The higher S index observed in older adults was primarily associated with shorter pre-heel-contact (pre-HC) STST duration, reduced total and single-support push-off impulses, and increased collision impulse during double support. Together, these findings indicate that older adults initiate STST later relative to push-off and rely more heavily on double-support force exchange, resulting in greater temporal overlap between push-off and collision. This shift in timing and impulse distribution likely limits effective redirection of the center of mass and contributes to reduced walking efficiency.

**Figure 5:**
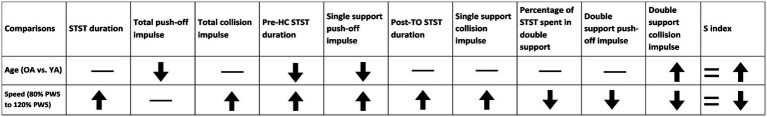
Summary of age- and speed-related changes in STST timing and impulse variables contributing to the S index. Arrows indicate direction of significant differences (↑ increase, ↓ decrease, — no change). The S index, reflecting STST efficiency, was *higher* in older adults due to decreased pre-HC duration, decreased total push-off impulse, lower single-support push-off impulse, and higher double support collision impulse, whereas S index was *lower* at faster speeds due to longer total STST, pre-HC, and post-TO durations, lower percentage of STST spent in double support, higher total collision impulse, lower double support push-off and collision impulses, and increased single support push-off and collision. Together, these findings illustrate how both timing and impulse components of STST drive age- and speed-related differences in STST efficiency.

Across both age groups, faster walking speeds were associated with improved STST efficiency, reflected by lower S index values. These improvements were supported by coordinated changes in both timing and impulse strategies, including longer total, pre-HC, and post-toe-off STST durations; reduced time spent in double support; increased total collision impulse; greater single-support push-off and collision impulses; and reduced double-support impulses. Notably, these speed-related adaptations were similar between young and older adults, suggesting that despite age-related deficits in baseline STST efficiency, both groups employed comparable mechanical strategies to improve efficiency at higher walking speeds (Figure 5).

### Walking speed and gait parameters

A notable feature of our present sample was the absence of significant differences in walking speeds, step rate, and step length and width between young and older adults. While this contrasts with literature reporting age-related reductions in PWS (Aboutorabi et al., 2016), similar null findings have been reported in studies of healthy older adults (Kang and Dingwell, 2008). Several factors may explain this result, for instance, our sample consisted of healthy, community-dwelling adults without pathology who required to be able to walk on treadmill at speeds faster than PWS, which may preserve speed capacity. Importantly, comparable walking speed and gat characteristics strengthen our mechanistic findings by allowing us to attribute observed S index differences to age-related changes in transition mechanics rather than to differences in locomotor demand.

### S index as a marker STST efficiency

A key finding of this study is that older adults consistently exhibited higher S index values than young adults across walking at PWS as well as at speeds slower and faster than PWS, with a medium effect size. Importantly, this age-related difference was replicated when walking speeds were matched across groups from 0.5 m/s to 1.7 m/s in a subset of participants who completed the matched-speed protocol (Appendix B). This replication demonstrates that higher S index values in older adults are robust and not an artifact of potential differences in PWS. Thus, even when both groups walk at identical absolute speeds, older adults demonstrate higher S index, suggesting greater energy loss and lower STST efficiency. This may contribute to the increased age-related energetic cost of walking. This outcome aligns with theoretical work by Ruina et al. (2005), that described how overlap between push-off and collision phases results in energy loss during walking. The S index quantifies this overlap, with higher values indicating greater simultaneous execution of positive (push-off) and negative (collision) work. Thus, our results confirm that older adults experience less efficient redirection of CoM during STST. By incorporating both the timing and magnitude of opposing ground reaction forces during step transitions, the S index provides a mechanical proxy for energy losses that must ultimately be offset by metabolic energy expenditure. This interpretation is consistent with collision-based models of walking energetics that link inefficient push-off–collision coordination to increased step-transition work and metabolic cost (Donelan et al., 2002a,b; Soo and Donelan, 2012). As such, the S index represents a promising metric for assessing gait efficiency using step-level ground reaction force data alone, offering an alternative to traditional approaches that rely on metabolic measurements and extended data collection. Future work should examine the direct relationship between S index, metabolic cost, and established measures of gait efficiency to further evaluate its utility as an indicator of walking efficiency across populations.

Previous studies have consistently demonstrated a U-shaped relationship between walking speed and energetic cost, with the minimum energy expenditure occurring at approximately 1.1–1.3 m/s (average PWS in humans) in both young and older adults (Mian et al., 2006; Ralston, 1958; Schrack et al., 2013, 2016). Although, this fundamental relationship is preserved with aging, the entire cost-speed curve is shifted upward in older adults, indicating higher metabolic energy expenditure at all walking speeds. Previous work (Mian et al., 2006) has attributed this elevated cost to lower overall efficiency in older adults. Our findings provide mechanistic insight into this phenomenon by showing that as older adults demonstrate persistently lower step-to-step transition efficiency (higher S index) across all speeds, even though the step-level improvement in S index with speed follows the same directional pattern as younger adults. Importantly, although the S index improves with increasing speed in both age groups, older adults remain consistently less efficient than young adults. This suggests that while older adults retain the ability to modulate push-off and collision sequencing in response to speed demands, age-related neuromuscular and temporal constraints limit their ability to achieve the transition efficiency observed in younger adults (Jeon et al., 2025; Mathieu et al., 2024; Summerside et al., 2024). Furthermore, the preserved speed-dependent modulation of the S index indicates that STST efficiency may be amenable to targeted interventions aimed at improving push-off generation and the timing and coordination of push-off and collision forces.

### Neuromechanical strategies underlying STST efficiency

While the primary analyses demonstrated age-related differences in S index as a marker of step-to-step transition (STST) efficiency, further insight was gained by decomposing the S index into its constituent timing and impulse components. Specifically, examining total STST duration, pre-HC, post-TO durations, and percentage of STST spent in double support and impulse components (push-off and collision impulses), allowed us to identify neuromechanical strategies that underlie age- and speed-related changes in STST efficiency. This decomposition highlights specific temporal and force-generation mechanisms that likely contribute to inefficient CoM redirection in older adults and identifies distinct variables that may be targeted to improve walking efficiency through intervention (Figure 5).

The higher S index observed in older adults was associated with shorter pre-HC STST duration, lower total push-off impulse, higher double support collision impulse, and reduced single support push-off impulse while there were no significant changes in total STST and post-TO durations, percentage of STST spent in double support, and double support push-off, single support collision, and total collision impulses. These results align with and extend a growing body of literature indicating that multiple neuromechanical pathways contribute to age-related changes in STST control.

STST can be executed via two distinct neuromechanical strategies: an active mode that starts before the initial foot contact, involving anticipatory central nervous system control, and a passive mode that relies more heavily on mechanical properties and impact forces at foot contact (Winter, 1991). Prior studies have shown that young adults typically employ active STST strategies, whereas older adults tend to exhibit characteristics of more passive STST, with greater reliance on double-support collision forces (Chong et al., 2009; Dewolf et al., 2019, 2022; Meurisse et al., 2019a). Our observation of shorter pre-HC STST duration and higher double support collision impulse in older adults aligns with this body of work. However, rather than viewing this pattern solely as consequence of deteriorating neuromuscular capacity, it may alternatively reflect an adaptive recalibration of motor control. Several lines of evidence support an adaptive control interpretation: First, our results demonstrate that older adults successfully modulate their STST strategies with walking speed, achieving lower S index values at higher speeds through systematic increases in single-support impulses and decreases in double-support overlap. This capacity to shift toward more efficient coordination patterns as task demands increase indicates that the underlying motor system remains responsive and adaptable, rather than fundamentally constrained.

Second, intervention studies have demonstrated that older adults can improve walking efficiency and reduce energetic cost through targeted training of push-off timing and stepping coordination (Collins et al., 2018; Franz et al., 2014; VanSwearingen et al., 2009, 2011). These findings suggest that lower efficiency often observed at preferred walking speed may reflect a learned or habitually-adopted strategy rather than a fixed neuromuscular capacity limitation. Third, recent work has shown that older adults are capable of adopting new movement strategies that either enhance efficient walking or deliberately trade energetic efficiency for balance maintenance, depending on task constraints and behavioral priorities (Jeon et al., 2025; Mathieu et al., 2024; Summerside et al., 2024).

Taken together, these findings suggest that the observed alterations in STST timing and impulses, including shorter pre-HC duration, lower total push-off impulse, higher double support collision impulse, and reduced single support push-off impulse, may represent adaptive recalibration of motor control. This recalibration appears to be indirectly shaped by age-related neuromuscular constraints (such as reduced propulsive power, slower force generation capacity, and altered proprioceptive feedback) but is not solely dictated by these constraints. From a motor control perspective, these changes can be interpreted as a reweighting of control strategies under neuromuscular constraints. Increased reliance on double-support impact forces and delayed push-off may reduce the need for rapid, high-magnitude propulsive muscle activation, potentially contributing to greater stability, while coinciding with reduced step-to-step transition efficiency.

The role of neuromuscular decline should not be minimized; reduced capacity to generate rapid plantarflexor torque, declining propulsive power, and impaired anticipatory postural adjustments certainly constrain the available motor strategies. However, the specific pattern of altered STST timing and impulses observed in older adults may represent the nervous system’s functional solution for locomotion under these constraints, rather than simply an inbaility to implement t efficient patterns. Understanding the gait patterns of older adults as potentially adaptive, rather than purely deficit-based, opens new possibilities for intervention design, where the goal would be to expand the constraint envelope and enable access to more efficient strategies, rather than simply attempting to restore young-adult-like patterns.

Prior research has shown that age-related changes in gait and balance are more pronounced in older adults who initiate the step-to-step transition (STST) at or after heel contact, compared with those who initiate STST earlier (Meurisse et al., 2019b). One potential contributor to these timing differences is an age-related impairment in Anticipatory postural adjustments, a feedforward control mechanism to enhance balance prior to perturbations (Kanekar and Aruin, 2014). Accordingly, the shorter pre-HC STST duration in older adults may partly reflect diminished anticipatory control. Further, the ability to generate rapid plantarflexor torque declines with age (Thelen et al., 1996). The lower total and single support push-off impulses observed in our study may be due to both a decline in rapid torque generation and a shortening of the available time window for propulsive force. Indeed, our study showed that older adults had a shorter single support push-off phase during STST, compounding the limitation in torque generation. Together, these findings suggest a dual constraint: older adults not only have reduced force-generating capacity but also have less available time to apply force generation during the propulsive phase. These constraints shape the space of feasible motor control solutions available to the nervous system. How older adults allocate timing and impulses during STST may reflect adaptive motor control rather than an inability to produce efficient patterns (Jeon et al., 2025; Mathieu et al., 2024; Summerside et al., 2024).

The altered STST timing, characterized by shorter period spent in pre-HC single support, is likely to contribute to the lower efficiency observed in older adults. Specifically, shorter single-support phases have been shown to lead to lower propulsive impulses and greater reliance on double-support, where more energy is used due to simultaneous push-off and collision forces (Ruina et al., 2005; Van Beek et al., 2021). Consistent with this framework, studies of STST timing indicate that initiating push-off prior to heel contact and extending STST beyond toe-off can reduce collision losses and improve walking efficiency. Younger adults typically employ this sequencing, effectively minimizing overlap between opposing forces and achieving lower S-index values (Adamczyk and Kuo, 2009). Conversely, older adults, who often initiate STST later and end it earlier, incur higher energy loss due to increased overlap between opposing forces. The functional importance of push-off timing is further supported by intervention studies showing that biofeedback or targeted training can improve push-off timing in older adults (Franz et al., 2014), resulting in increased preferred walking speed and reduced energetic cost of walking (Collins et al., 2018; VanSwearingen et al., 2009, 2011). Age-related changes in coordination and timing of STST initiation and completion (Alijanpour and Russell, 2025) can impair the ability to redirect the CoM effectively during STST, thereby contributing to the increased metabolic cost of walking (Franz et al., 2014; Collins et al., 2018; VanSwearingen et al., 2009, 2011).

Both young and older adults exhibited lower S index at higher speeds, driven by increased single-support push-off and collision impulses, and reduced double-support impulses. Total STST duration increased across speeds through longer pre-HC and post-TO durations, while percentage of STST spent in double support decreased. This reallocation of STST phases toward greater single-support engagement is consistent with prior findings showing enhanced gait efficiency at higher speeds through improved muscle force timing and neuromuscular control (Clark et al., 2013; Núñez-Lisboa and Dewolf, 2025). Critically, the fact that both age groups successfully implement these more efficient STST strategies when walking speed increases suggests that the motor capacity required to achieve lower S index values is preserved in older adults, even though these patterns are not typically expressed at PWS. This raises an important question: if older adults can achieve lower S index at higher speeds, why do they not spontaneously adopt these more efficient patterns at their preferred speed? Several not mutually exclusive possibilities warrant consideration. First, preferred walking speed may emerge from an optimization process that weighs multiple competing objectives, not only energetic cost, but also stability, safety, and cognitive load. The greater reliance on double-support and delayed single-support push-off observed at preferred speed in older adults may represent a conservative strategy that prioritizes balance maintenance and fall prevention over minimizing step-to-step transition costs. Second, the speed-dependent improvements in STST efficiency may reflect task-driven constraints: at higher speeds, the greater mechanical demands may necessitate improved neuromuscular coordination to maintain stability, thereby revealing capacities that are less engaged at preferred speed. Third, the alterations in STST at preferred speed may reflect long-standing adaptive adjustments that have become habitual or “learned,” potentially involving not only motor output but also proprioceptive recalibration and changes in motor planning. These possibilities suggest that understanding the preferred walking speed of older adults requires integrating biomechanical, neuromuscular, and motor control perspectives, not attributing it solely to capacity limitations. Taken together, these considerations suggest that understanding preferred walking speed in older adults requires integrating biomechanical, neuromuscular, and motor control perspectives, rather than attributing age-related differences solely to limitations in motor capacity.

This study is the first to investigate S index values in older adults’ gait and across various speeds. It is a preliminary effort to provide insight into the potential of S index as a representative of gait efficiency at the level of individual steps. Several limitations should be considered when interpreting our findings. First, our study focused exclusively on healthy, community-dwelling adults with no major health issues and clinical conditions. This likely underrepresents the broader spectrum of older adult gait variability, especially among individuals with clinical impairments or elevated fall risk and may bias results toward higher-functioning older adults. Further, while treadmill-based gait data offer precise kinematic and kinetic control, they may not fully reflect overground walking dynamics (Ojeda et al., 2015). Prior work has shown that treadmill walking can fundamentally alter locomotor strategies; for example, Ojeda et al. (2015) demonstrated a reversal in the covariation of stride length and speed on a treadmill compared to overground walking, consistent with predictions by Dingwell and colleagues. Such findings highlight that treadmill constraints may elicit different neuromotor control strategies, which is particularly relevant when studying older adults who may adapt differently to non-ecological surfaces. Complementing treadmill findings with overground data in future studies will provide more ecologically valid insights into age-related gait adaptations.

## Conclusion

In summary, using the S index as step-level metric, this study offers novel insights into mechanisms underlying age-related changes in STST efficiency. Across all walking speeds, older adults exhibited significantly higher S-index values than young adults, indicating greater temporal overlap between push-off and collision forces and, consequently, reduced mechanical efficiency of STST. These higher S-index values were accompanied by shorter pre-HC duration, lower total push-off impulse, higher double support collision impulse, and reduced single support push-off impulse. While these mechanical and temporal characteristics are certainly influenced by age-related neuromuscular changes, such as reduced propulsive power, slowed force generation capacity, and altered proprioceptive feedback, they may also reflect adaptive recalibration of motor control strategies (Jeon et al., 2025; Mathieu et al., 2024; Summerside et al., 2024). The fact that older adults successfully implement more efficient STST patterns when walking speed increases suggests that the altered patterns observed at preferred walking speed may represent controlled trade-offs between energetic efficiency and other objectives (such as stability and balance maintenance) rather than purely capacity-limited deficits. Collectively, these findings highlight the S index as a step-specific, mechanically grounded metric of gait efficiency that captures age-related deficits in propulsion, timing, and motor coordination. Future work should further evaluate the utility of the S index by linking it to metabolic cost, muscle activation patterns, and neurophysiological measures, with the goal of clarifying how step-level control strategies contribute to locomotor efficiency and its decline with aging.

## Data Availability

The original contributions presented in the study are included in the article/Supplementary material, further inquiries can be directed to the corresponding author.
