# Supplementary material for: The Sequence (S) index as a marker of diminished step-to-step transition efficiency in older adults
Source: Front Hum Neurosci. 2026 Feb 25;20:1710840. doi: 10.3389/fnhum.2026.1710840 (PMC12979937; doi:10.3389/fnhum.2026.1710840)
Supplement: Supplementary file 1 [file Data_Sheet_1.docx]

**Appendix A: Individual participant values**

Appendix A. Table 1. Demographic characteristics of each participant


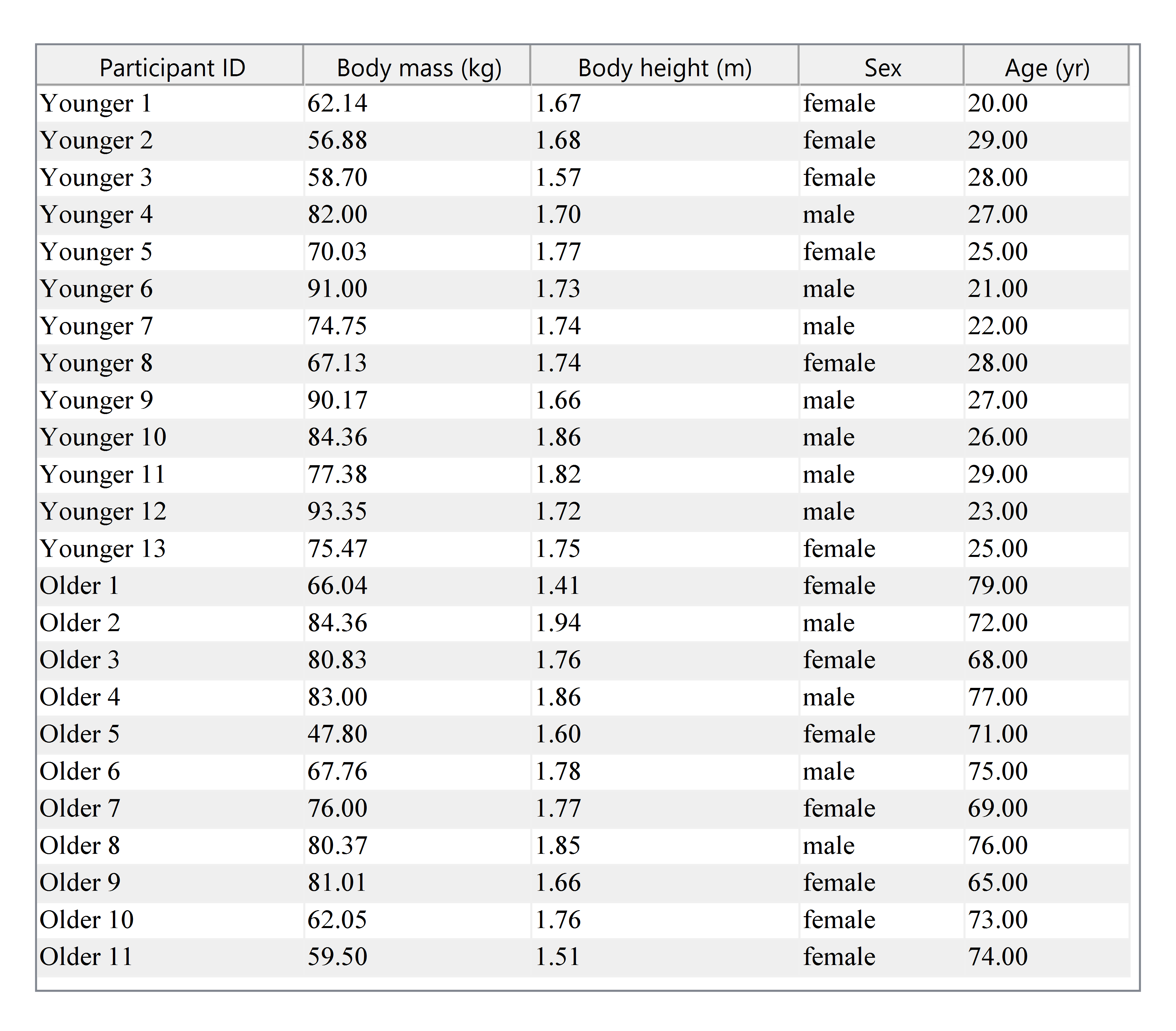


Appendix A. Table 2. Walking speed (m/s) information for each participant at each walking condition


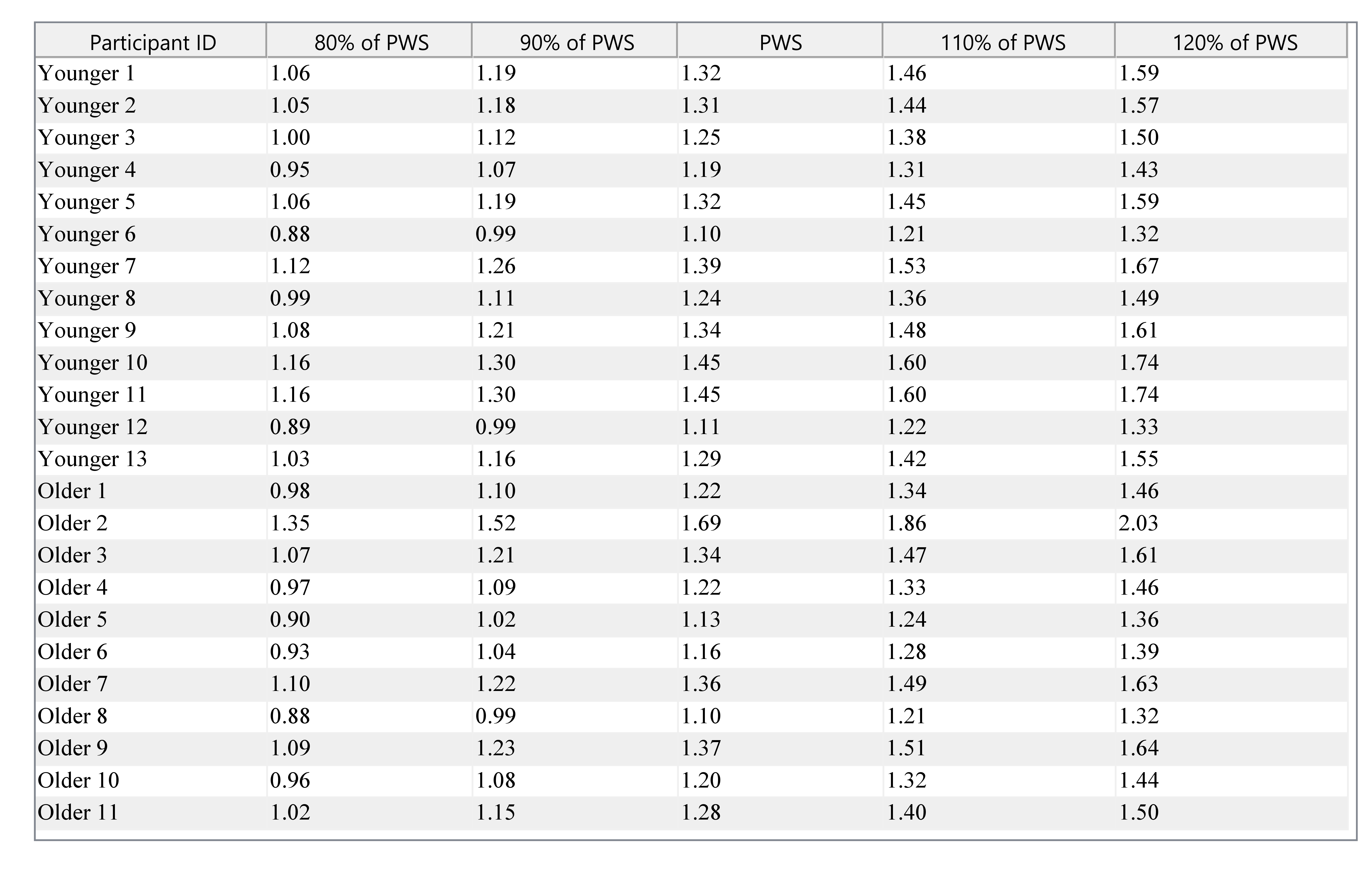


Appendix A. Table 3. S index values for each participant at each walking condition


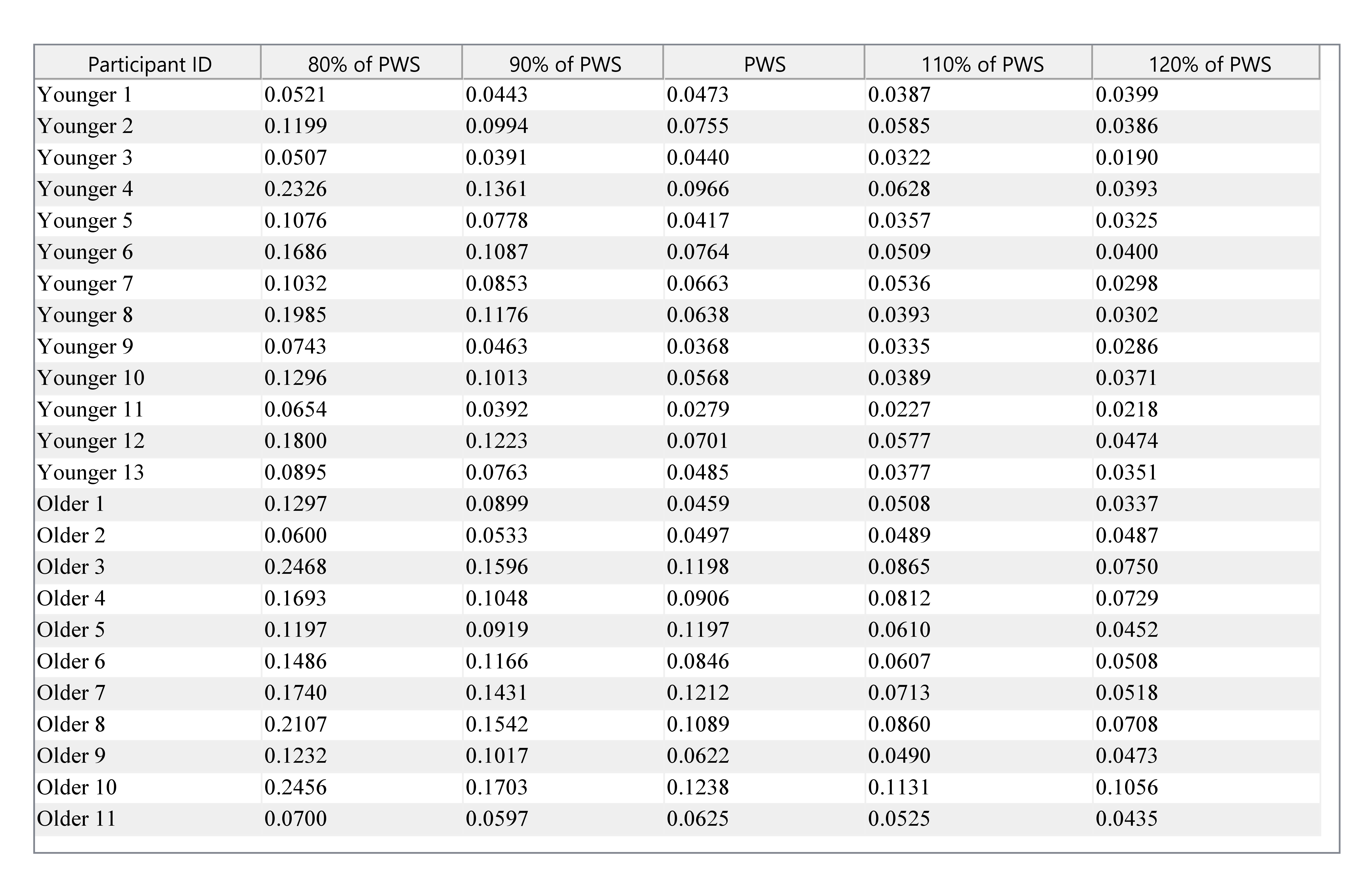


**Appendix B**

**
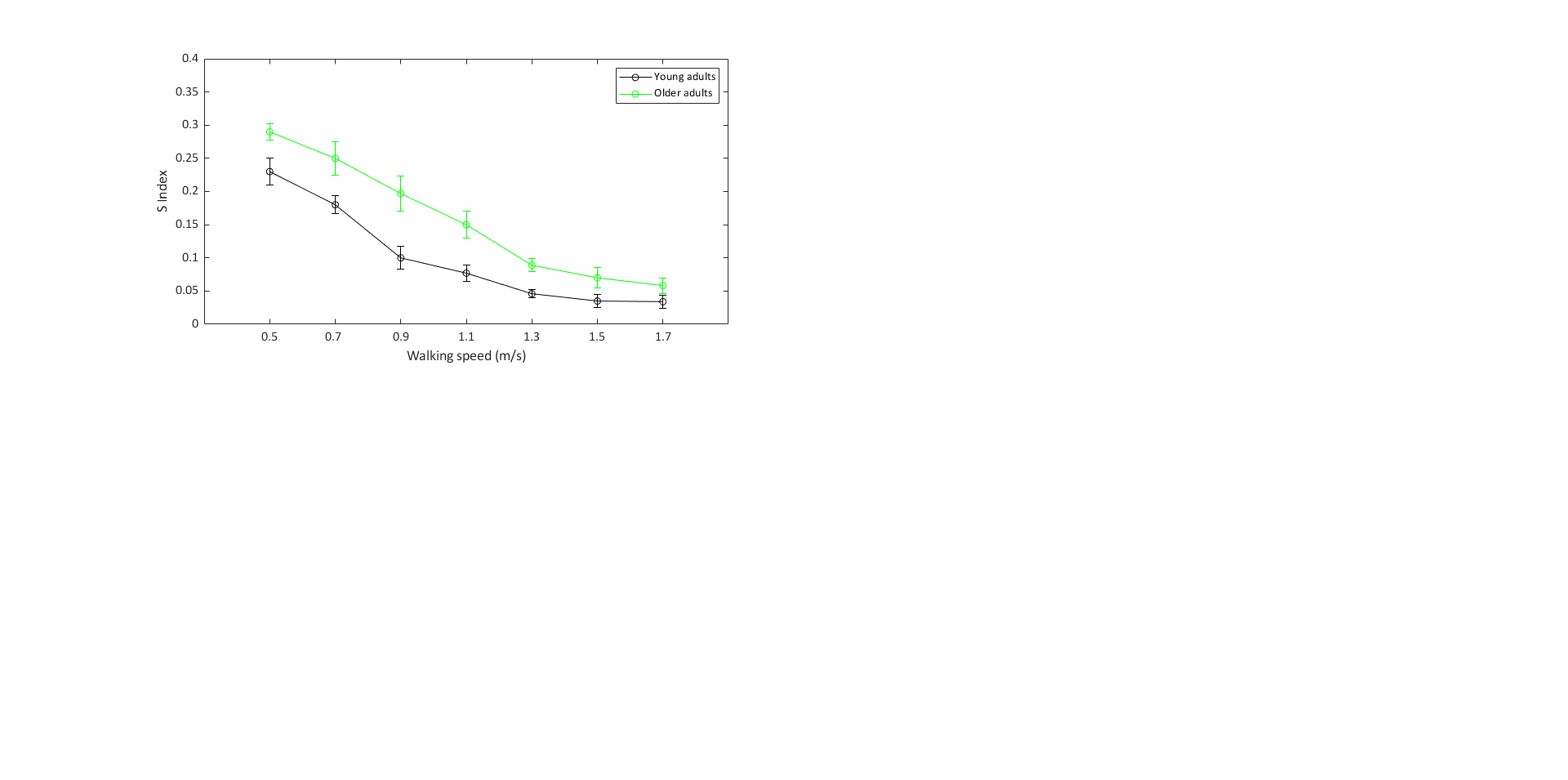
**

Supplementary figure 1. S index values for young (black) and older adults (green) walking at seven different speeds from slow to fast. The error bars indicate ±1 standard deviation.

To address the potential limitation of non-inferiority in preferred walking speeds between groups, we computed the S index from an additional treadmill protocol in which participants walked at fixed absolute speeds from 0.5 to 1.7 m/s (increments of 0.2 m/s). Please note that only seven participants in each group were able to complete this incremental task. This incremental test was a single trial in which participants walked for 30 seconds at each speed, the initial speed to start with was 0.5 m/s and it incrementally increased for 0.2 m/s until 1.7 m/s. Consistent with the main analysis, older adults exhibited approximately 0.06 higher S index values compared to younger adults across all speeds t(11.12) = 3.32, p <.001, b = 0.059, confirming reduced step-to-step transition efficiency. The trend of decreasing S index with increasing speed was observed in both groups, but older adults remained consistently less efficient. These results reinforce the robustness of our primary findings and support the conclusion that age-related increases in S index are present regardless of speed-scaling method.
